# Reticulated aspect!

**DOI:** 10.11604/pamj.2017.28.249.13020

**Published:** 2017-11-21

**Authors:** Amina Kissou, Badr Eddine Hassam

**Affiliations:** 1 Department of Dermatology, Ibn Sina Hospital, Rabat, Morroco

**Keywords:** Carteaud, gougerot, papillomatosis

## Image in medicine

A 25-year-old woman presented with a 5-year history of a reticulated pigmented rash on her upper chest and her back (A). The rash was asymptomatic, squamous and progressively spread to involve her axillae and her neck (B). There were no diabetes, no thyroid disorder. Previous courses of ketoconazole have been tried with no resolution. Scotch test was negative. Histology confirmed a diagnostic of confluent and reticulated papillomatosis of Gougerot and Carteaud (CRPGC). This patient was treated with 100mg a day of doxycycline for 04 months with good improvement. Confluent and reticulated papillomatosis of Gougerot and Carteaud was first described in 1927. With the help of electron microscopy, it has been elucidated that CRPGC arises due to aberrant keratinization. It is an uncommon condition that affects adolescents, especially women with dark skin, often no or misdiagnosed. The etiopathogenesis remains unknown. Clinically, the lesions are asymptomatics. The distrubition of these lesions are: facial, truncal, acral or flexular. This condition may be associated with obesity, diabetes, some endocrine disorders, genodermatosis and some drugs (lithium). Histology shows hyperkeratosis with focal parakeratosis, papillomatosis, moderate acanthosis and hyperpigmentation of the basal layer. Scotch test is negative. Although successful treatment with topical keratolytics, retinoids or antifungals has been reported; antibiotics, such as cyclines, at anti-inflammatory doses; have emerged as a preferred therapeutic option.

**Figure 1:**
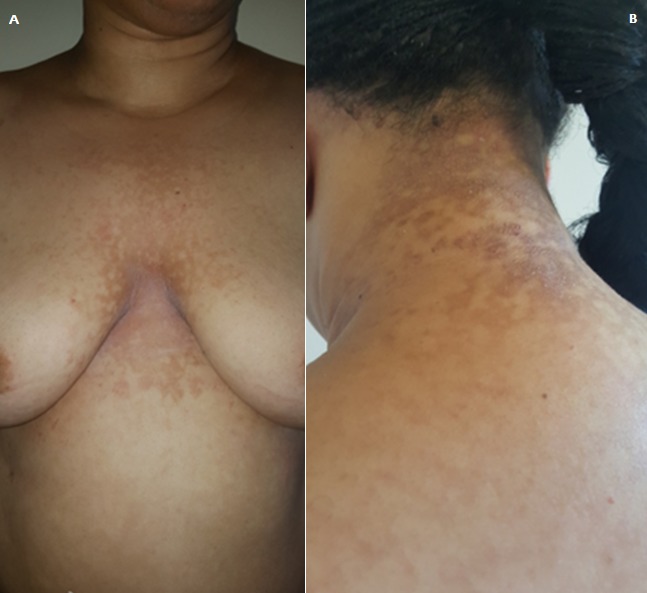
(A) reticulated pigmented rash on chest; (B) eticulated pigmented rash on the neck

